# Seed preferences by rodents in the agri‐environment and implications for biological weed control

**DOI:** 10.1002/ece3.2329

**Published:** 2016-07-22

**Authors:** Christina Fischer, Manfred Türke

**Affiliations:** ^1^ Restoration EcologyDepartment of Ecology and Ecosystem ManagementTechnische Universität MünchenEmil‐Ramann‐Str. 6D‐85354FreisingGermany; ^2^ Terrestrial Ecology Research GroupDepartment of Ecology and Ecosystem ManagementTechnische Universität MünchenHans‐Carl‐von‐Carlowitz‐Platz 2D‐85354FreisingGermany; ^3^ German Centre for Integrative Biodiversity Research (iDiv) Halle‐Jena‐LeipzigDeutscher Platz 5eD‐04103LeipzigGermany; ^4^ Institute of BiologyLeipzig UniversityJohannisallee 21D‐04103LeipzigGermany

**Keywords:** Cafeteria experiment, ecosystem disservice, ecosystem service, endozoochory, feeding preferences, post‐dispersal seed predation

## Abstract

Post‐dispersal seed predation and endozoochorous seed dispersal are two antagonistic processes in relation to plant recruitment, but rely on similar preconditions such as feeding behavior of seed consumers and seed traits. In agricultural landscapes, rodents are considered important seed predators, thereby potentially providing regulating ecosystem services in terms of biological weed control. However, their potential to disperse seeds endozoochorously is largely unknown. We exposed seeds of arable plant species with different seed traits (seed weight, nutrient content) and different Red List status in an experimental rye field and assessed seed removal by rodents. In a complementary laboratory experiment, consumption rates, feeding preferences, and potential endozoochory by two vole species (*Microtus arvalis* and *Myodes glareolus*) were tested. Seed consumption by rodents after 24 h was 35% in the field and 90% in the laboratory. Both vole species preferred nutrient‐rich over nutrient‐poor seeds and *M. glareolus* further preferred light over heavy seeds and seeds of common over those of endangered plants. Endozoochory by voles could be neglected for all tested plant species as no seeds germinated, and only few intact seeds could be retrieved from feces. *Synthesis and applications*. Our results suggest that voles can provide regulating services in agricultural landscapes by depleting the seed shadow of weeds, rather than facilitating plant recruitment by endozoochory. In the laboratory, endangered arable plants were less preferred by voles than noxious weeds, and thus, our results provide implications for seed choice in restoration approaches. However, other factors such as seed and predator densities need to be taken into account to reliably predict the impact of rodents on the seed fate of arable plants.

## Introduction

Various ecological processes can influence the fate of a seed, once it is released from the parent plant. Some processes may reduce the successful germination and seedling establishment; others may provide. While post‐dispersal seed predation can significantly reduce recruitment in many plant species (Kollmann 1995; Bricker et al. 2010; Maron et al. 2012; Crawley 2013; but see Pinto et al. 2014), seed dispersal can facilitate plant recruitment, for example, by decreasing intra‐ and interspecific competition and by increasing the probability of seeds to reach microsites suitable for germination (Cousens et al. 2008; Nathan et al. 2008; Pinto et al. 2014). Seed dispersal by animals can be mediated through deliberated transport of seeds to shallow caches (synzoochory; Cousens et al. 2008), adhesion of seeds to the body (epizoochory), or ingestion of seeds, if they are egested viable (endozoochory; Benvenuti 2007). To estimate the impact of animals on the post‐dispersal seed fate, as well as on plant population dynamics, both processes, either limiting recruitment by predation or facilitating recruitment by dispersal, have to be disentangled (Nathan and Muller‐Landau 2000; Vander Wall et al. 2005).

Seed predation has been discussed as an ecosystem disservice in agricultural landscapes, reducing the yield if crop seeds are consumed (Zhang et al. 2007; Schäckermann et al. 2015). Contrarily, seed predators that prey on seeds of noxious weeds can provide regulating ecosystem services in agricultural fields, resulting in biological weed control (Westerman et al. 2003; Daedlow et al. 2014). Seed dispersal is also discussed as an ecosystem service or disservice, depending on the target species. In the case of plant species, which lack obvious adaptations for dispersal, resulting in limited primary dispersal distances (Bischoff 2005), animal‐mediated seed dispersal possibly leads to a restoration of farmland biodiversity (Benayas and Bullock 2012). On the other hand, seed dispersal of common weeds can be an ecosystem disservice, spreading noxious species in agricultural fields (Liebman et al. 2001; Türke et al. 2013), thereby affecting crop production and increasing production costs (Zhang et al. 2007). As the seeds from a majority of plants growing in the agri‐environment lack adaptations for dispersal, and plants mostly rely on gravity for shedding their seeds (Benvenuti 2007), endozoochory might be an important, but rarely investigated mechanism of dispersal (Türke et al. 2013).

Both processes, seed predation and endozoochorous seed dispersal, are shaped by seed consumers’ identity and density (cf. Will and Tackenberg 2008), their behavior, such as the visitation rate of food patches and feeding duration (Cousens et al. 2008, 2010) or the predation risk during foraging (Nonacs 2001; but see Birthisel et al. 2014), as well as by seed morphology, such as seed size, weight, and nutrient content (Honek et al. 2007; Booman et al. 2009; Hintze et al. 2013). Seed predation rates are further influenced by the familiarity of seed consumers with food items (Crawley 2013) and weed species identity (Fischer et al. 2011), the abundances of different seed species and apparent competition (Abrams and Matsuda 1996; Schartel and Schauber 2016); but also by environmental factors such as vegetation cover, which can increase consumption rates (Meiss et al. 2010). Post‐dispersal seed predation and endozoochorous seed dispersal are mainly caused by generalist species, such as rodents and granivorous birds (Vander Wall et al. 2005; Crawley 2013), but also by invertebrates (e.g., Honek et al. 2007; Türke et al. 2013). With regards to endozoochorous seed dispersal, certain seed traits, in particular, an impermeable, hard seed coat and small seed size, enhance seed survival (Cousens et al. 2008).

Small rodents abundantly occur in agricultural landscapes (Heroldová et al. 2007; Fischer and Schröder 2014) and can be important seed predators (Westerman et al. 2003; Fischer et al. 2011; Daedlow et al. 2014). Seed predation by small rodents depends on seed weight and nutrient content (Wang and Yang 2014), their metabolic requirements, and their feeding behavior (Butet and Delettre 2011). In contrast, the knowledge about the role of small rodents facilitating secondary dispersal of weed seeds is limited (Vander Wall et al. 2005; Benvenuti 2007). Endozoochorous seed dispersal of small‐seeded species (<1 mg) was observed in invasive rats (Williams et al. 2000; Bourgeois et al. 2005; Shiels and Drake 2011). However, nothing is known about the potential of rodents in agricultural landscapes, which differ strongly in behavior and morphology from rats, to disperse seeds endozoochorously.

To elucidate small rodents’ seed feeding behavior, depending on different seed traits, as well as their ecosystem functions as seed predators and/or endozoochorous seed dispersers, we studied the impact of small rodents on the post‐dispersal seed fate of arable plant species. Feeding preferences for plants with different seed traits (lightweight vs. heavy seeds; nutrient‐rich vs. nutrient‐poor seeds), and with different occurrence probability (common vs. endangered plant species), as well as seed removal rates, and the endozoochorous seed dispersal potential of small rodents were assessed in field and laboratory experiments to answer the following questions:

1. What are the most important seed and plant traits (seed size and nutrient content, plants’ occurrence probability) influencing small rodents feeding preference and seed removal/predation rates?
2. Are small rodents seed predators rather than endozoochorous seed dispersers of arable plant species in agricultural landscapes?

## Material and Methods

### Seed species and seed characteristics

In total, seeds of 15 arable plant species which generally reproduce by seed (Table S1 in Supporting Information), including common and endangered plants, were tested for seed consumption by small rodents (Table 1). The twelve common plant species are regularly growing in arable fields, field margins, and ruderal habitats and can be harmful for crop production (Hofmeister and Garve 2006). *Galium aparine,* for instance*,* has the lowest economic threshold level among the species in our set, with 0.1 plants·m^−^² (Table 1), and it can overgrow cereal crops, favor fungal diseases, and cause a high yield loss (Gehring and Thyssen 2011). The three endangered plant species in our set are listed in the Red List of endangered plants in Bavaria (category 3, StMUGV 2005) and are also rare or threatened across Europe (Storkey et al. 2012). Seeds of the common species were supplied by Appels Wilde, Darmstadt, and seeds of the endangered species were supplied by a local seed producer (J. Krimmer, Pulling).

**Table 1:** Arable plant species (nomenclature according to Wisskirchen and Haeupler 1998) which were used for the seed removal/predation experiment in the field and in the laboratory, including seed traits and occurrence probability (Red List status and economic threshold level, wherever available). For the field experiment, removal rates by rodents after 24 h (SR_R_), and for the laboratory experiment, predation rates for both tested vole species (Microtus arvalis or Myodes glareolus) after 6 h (SP_R_) and Rodgers’ preference indices (*R*_i_) for cafeteria experiments are shown

Plant species	Trait			Field		Laboratory			
	Weight class	Nutrients^a^ (1 = yes, 0 = no)	Red list status^b^/Economic threshold level (plants·m^−^²)^c^	SR_R_ (%)		SP_R_ (%)		*R*_i_	
				“normal” sowing rate	“reduced” sowing rate	*M. arvalis*	*M. glareolus*	*M. arvalis*	*M. glareolus*
*Alopecurus myosuroides* Huds.	Light	1	Common (15–30)	–		50.0 ± 22.4	63.0 ± 9.7	0.6 ± 0.1	0.7 ± 0.1
*Apera spica‐venti* (L.) P. Beauv	Light	1	Common (10–30)	–		46.7 ± 10.2	63.5 ± 7.8	0.6 ± 0.1	0.7 ± 0.0
*Buglossoides arvensis* (L.) I. M. Johnst.	Heavy	0	Endangered (3)	62.2 ± 5.6	26.4 ± 10.6	26.7 ± 13.3	11.5 ± 2.8	0.5 ± 0.1	0.2 ± 0.0
*Capsella bursa‐pastoris* (L.) Med.	Light	0	Common	–		16.7 ± 9.9	51.0 ± 7.8	0.4 ± 0.1	0.6 ± 0.0
*Cirsium arvense* (L.) Scop.	Light	0	Common	30.3 ± 6.3	47.0 ± 10.2	21.7 ± 10.1	71.0 ± 9.5	0.5 ± 0.1	0.7 ± 0.1
*Consolida regalis* Gray	Light	0	Endangered (3)	45.4 ± 9.3	20.0 ± 6.1	31.7 ± 12.0	66.0 ± 9.7	0.5 ± 0.1	0.8 ± 0.1
*Elymus repens* (L.) Gould s. str	Heavy	1	Common	–		51.7 ± 12.5	39.0 ± 8.0	0.6 ± 0.1	0.5 ± 0.0
*Galium aparine* L.	Heavy	0	Common (0.1)	39.9 ± 8.4	25.4 ± 5.4	23.3 ± 5.6	21.0 ± 5.2	0.4 ± 0.0	0.3 ± 0.0
*Legousia speculum‐veneris* (L.) Chaix	Light	0	Endangered (3)	50.6 ± 7.9	19.2 ± 7.0	40.0 ± 15.3	57.5 ± 8.5	0.5 ± 0.1	0.6 ± 0.1
*Matricaria recutita* L.	Light	0	Common	–		10.0 ± 8.2	66.5 ± 8.9	0.4 ± 0.0	0.7 ± 0.1
*Poa trivialis* L. s.l.	Light	1	Common (50 *Poa annua*)	–		21.7 ± 10.5	66.5 ± 9.4	0. 5 ± 0.1	0.7 ± 0.1
*Stellaria media* (L.) Vill. s.str.	Light	0	Common (25)	43.1 ± 10.6	44.1 ± 10.9	33.3 ± 3.3	66.0 ± 8.9	0.5 ± 0.0	0.7 ± 0.1
*Thlaspi arvense* L.	Light	0	Common	28.9 ± 6.7	15.8 ± 5.7	21.7 ± 15.8	66.0 ± 10.0	0.4 ± 0.1	0.7 ± 0.1
*Tripleurospermum perforatum* (Mérat) Lainz	Light	0	Common (3–5)	–		46.7 ± 13.1	61.0 ± 8.6	0.6 ± 0.1	0.7 ± 0.1
*Viola arvensis* Murray	Light	1	Common (5)	45.3 ± 9.4	21.1 ± 6.7	30.0 ± 7.3	67.5 ± 8.6	0.5 ± 0.0	0.7 ± 0.1

a Traits extracted from the D^3^ database (Hintze et al. 2013).

b Occurrence probability and Red List status (3: endangered) extracted from the Red List of endangered plants in Bavaria (StMUGV 2005).

c Economic threshold level for noxious weeds in cereal grains, defined as plants·m^−^² (Gehring and Thyssen 2011).

Seed weight was either measured by weighing seeds in groups of ten, with ten replicates per species and calculating the mean weight per seed in mg, or it was retrieved from the “Dispersal and Diaspore Database” (D³: Hintze et al. 2013) or the “Seed Information Database” (SID: Royal Botanic Gardens Kew 2015; Table S1). As seed weight of the 15 species was highly skewed, with a higher number of small‐seeded species, we converted the numeric variable “seed weight” into a factor with the two classes “light” and “heavy” with a threshold level of 3.62 mg using the “cut” command in R 3.0.2 (R Core Team 2013). For the nutrient content, the binary variable presence (1) with diaspores containing a significant amount of nutrients in quality or quantity, or absence (0) was used (Hintze et al. 2013). Hintze et al. (2013) defined this category as an indicator for dysochory and endozoochory, with nutrient‐rich seeds being more attractive for animal consumption than nutrient‐poor seeds.

### Field experiment

Seed removal was studied on an experimental crop field SW of Munich, Germany (N 48°7′43.149, E 11°25′1.469), which has been managed organically since the 1980s. The mean annual temperature and precipitation for the study area between 1981 and 2010 were 8.7°C and 834 mm (DWD 1981) and 9.2°C and 735 mm for the study year 2012. During the study period in June and July 2012, mean temperature was 17.9 ± 0.6°C and mean precipitation was 105.8 ± 19.8 mm (DWD 2012). The experimental field was established on an area of 198 × 53 m, surrounded by mixed forests, hedges, and a highway. The field was divided into five replications with 16 plots of 5.2 × 6.5 m each (in total 80 plots). Replications were separated by regularly mown grass verges of 6 m width. For the seed removal experiment, four plots per replication, which were randomly distributed within replication, were chosen, with a minimum distance of 6 m between each other. Two of the four plots were sown with winter rye with 350 seeds·m^−^², which corresponds to “normal” sowing rates in organic fields of the region, and two plots were sown with 88 seeds m^−^², which corresponds to “reduced” sowing rates, with a quarter of the usual seed numbers (Fig. S1). For further experiments, the three endangered plant species were sown in each plot to test for their ability to establish under different crop sowing rates (details in Prestele et al. 2013).

The seed removal experiment was conducted twice, in mid‐June and again in mid‐July 2012, by offering seeds of eight arable plants (Table 1), which also occurred in the field. Ten seeds of each species were offered at the same time on a 10 × 10 cm wooden plate (seed depot), which was divided into eight trays of 2 × 2 cm. Each seed species was randomly allocated to one of the trays. Trays were surrounded by a wooden barrier (0.5 × 0.3 cm w  ×  h) to avoid mixing of seeds of the different plant species or disruption by wind or animals walking over the seed depots. Barriers did not influence rodents’ feeding behavior or restrict the accessibility to certain seed species (proofed by video observations). One unprotected seed depot with access to all animals (“all access”) and one depot protected against rodents by a 125 × 125 × 40 mm (l × w × h) cage with a rhombus‐shaped mesh of 28 × 10 mm size (“no rodent access” proofed by Türke et al. 2010, 2012) were placed in each plot with a distance of 4 m between each other (in total 40 seed depots per trial). Seeds were exposed to predators in the field for 24 h due to high removal of some seed species. Furthermore, the observation period was adapted to video observations, which were constrained by battery runtime, as well as to the observation time of the laboratory experiment. Remaining seeds were counted and inspected for further seed damage. Seed removal by rodents (SR_R_ in %) was calculated following Fox et al. (2013), with SR_R_ = ((R_NRA_ − R_AA_)/R_NRA_) *100 [%], where R_NRA_ is the number of seeds remaining on the “no rodent access” depot, and R_AA_ is the number of seeds remaining on the “all access” depot on the same plot. Thereby, the fraction of seed removal by invertebrates was assumed to be equal in the “all access” and “no rodent access” treatment. In case R_NRA_ exceeded R_RAA_ by more than 5:4, the data point was removed from the analysis (Fox et al. 2013); in all other cases, seed removal was set to 0% (Saska et al. 2008). Mean values of both sampling rounds were calculated (c.f. Fischer et al. 2011).

To measure small rodents’ abundance and species composition on the experimental field, a capture–mark–recapture approach using 160 Ugglan multiple capture live traps (240 × 60 × 90 mm; Grahnab, Gnosjo, Sweden) was conducted. In each plot, two traps were placed in 0.5 m distance from the border, at opposing sites, with the opening showing to the interior (Fig. S1). Trapping was carried out in the beginning of July, between the two trials of the seed removal experiment. Rodent trapping was conducted following Fischer and Schröder (2014). Rodent abundance was calculated as the total number of individuals, excluding recaptures.

To confirm the identity of seed feeding rodents, we used weather protected video cameras (Panasonic WV‐BP122E) for three consecutive days parallel to the seed removal experiment. Five cameras were installed simultaneously, one camera in one additional plot with “reduced” sowing rate in each of the five replications. Cameras were placed approximately 17 cm from an “all access” seed depot and recorded nonstop over a period of 24 h, using a single infrared diode (LED; 880 nm, 55°) for observations during the night. After 24 h, cameras and depots were shifted to another plot within replicates and seeds were replenished. Videos were screened at eight times speed unless rodents were observed. Rodents feeding on seeds or removing seeds from depots, the duration of stay, and the feeding rate per seed species were identified. Feeding diversity (Shannon's index *H*) and evenness (*J*) from the eight seed species was calculated for voles and mice.

### Laboratory experiment

Voles were captured from wild populations on a fenced fallow land W of Freising, Germany (N 48°24′20.548, E 11°41′16.952) with an area of 70 × 30 m, surrounded by arable fields and mixed forest. Trapping was carried out on a site in proximity of the laboratory rather than on the more distant site used for the field experiment, to reduce the stress of transportation. In addition, we did not want to manipulate the density of the rodents in our experimental field. Trapping was carried out between mid‐June and mid‐July 2013 using Ugglan live traps. Six adult *Microtus arvalis* (Pallas) (weight: 22.5 ± 2.5 g mean ± SE; 3:3 females:males) and 20 *Myodes glareolus* (Schreber) (weight: 21.8 ± 0.8 g mean ± SE; 8:12 females:males) were used for a cafeteria experiment, excluding pregnant females. Both species are common in agricultural landscapes (Heroldová et al. 2007) and are of low or intermediate trophic position, mainly feeding on plant material, including seeds (Butet and Delettre 2011). Voles were brought to the laboratory, individually kept in fauna boxes (300 × 195 × 205 mm l × w × h), and not interfered for 24 h.

To test for feeding preferences, seeds of the same eight plant species used in the field experiment plus seven additional common weeds were offered to single vole individuals (Table 1). Plant species were randomly allocated to one of 15 trays (2 × 2 cm) on a 10 × 17 cm seed depot. Ten seeds per species were offered to the voles simultaneously. Feeding events were observed directly, and the number of consumed seeds was recorded every 15 min during the first 2 h, and every 30 min during the following 4 h. After 24 h, a final inspection was made, and all remaining seeds were collected and checked for feeding traces.

Seed predation rates were calculated from the initial number of seeds for each time step and seed species (SP_R_ in %). Feeding preferences were calculated by the Rodgers’ index (*R*_i_) for cafeteria experiments (Rodgers and Lewis 1985; Krebs 2014). Therefore, we calculated the area under the cumulative consumption curves over time per individual (*A*_i_) using the trapezoidal integration function from the R package pracma (Borchers 2015), standardized to a maximum *R*_i_ from all seed species and vole individuals (max(*A*_i_)) by: *R*_i_ = *A*_i_/max(*A*_i_). Preference scores range from 0 to 1 with 1 the seed species preferred most and those with a smaller *R*_i_ are less preferred (Rodgers and Lewis 1985; Krebs 2014).

To test for endozoochorous seed dispersal by voles, all feces per individual were collected from the fauna boxes after further 48 h, which is related to the gut passage time of seed diet by *Myodes* and *Microtus* spp. (Lee and Houston 1993). Thereafter, voles were marked by fur clipping to avoid reuse of individuals and were released at the place of capture. During the experiment, voles were handled in accordance to the Directive 2010/63/EU of the European Parliament and of the Council on the protection of animals used for scientific purposes (The European Parliament and the Council of the European Union 2010).

### Seed recovery after gut passage and germination

Collected feces from the laboratory experiment (*n* = 26) were softened overnight in 2‐ml tubes filled with tap water. Then, samples were gently homogenized by shaking vials and rinsed under running tap water, using a funnel suited with filter paper. Samples were searched for remaining seeds and their number and the seed species was registered. Finally, the complete sampling material per individual was evenly dispensed on moistened filter paper in Petri dishes. To compare germination rates of digested seeds with undigested seeds, 2 × 40 undigested seeds per species were put on moistened filter paper in Petri dishes. All samples were placed in a germinator with 12 h of light, 24°C and 12 h of dark, 15°C for 6 weeks. Samples were checked every 3 days, and germinated seeds were counted. Germination rates were calculated as the percentage of germinated seeds of the number of seeds tested.

### Statistics

For all analyses, R version 3.0.2 (R Core Team 2013) was used. For the field experiment, SR_R_ in relation to the different seed species, sowing rates of winter rye, and the two‐way interaction were tested using linear mixed effects models (lme; Pinheiro and Bates 2000) with a maximized log‐likelihood implemented in the R package nlme (Pinheiro et al. 2013). Impacts of seed weight (light vs. heavy), nutrient content (0 vs. 1), and occurrence probability (common vs. endangered) on SR_R_ were tested using separate lmes. The factors replication (*n* = 5) and plot (*n* = 20) nested within replication were included as random effects to model the independence of errors with respect to autocorrelations (Pinheiro and Bates 2000).

For the laboratory experiment, we first tested for differences in SP_R_ between the two vole species (*M. arvalis* and *M. glareolus*). Then, SP_R_ after 6 h (as almost all seeds had been consumed after 24 h) and *R*_i_ for each vole species were analyzed in relation to seed species, seed weight, nutrient content, and occurrence probability used as explanatory variables in separate lmes. The factor individual (*n*_*M. arvalis*_ = 6, *n*_*M. glareolus*_ = 20) was included as random effect to account for feeding preferences of different seed species by the same individual (Pinheiro and Bates 2000).

For all models, different variance structures were used where necessary to avoid heteroscedasticity (Pinheiro and Bates 2000). Model simplification was performed in a backward stepwise model selection procedure by AIC (Akaike's Information Criterion) implemented in the R package MASS (Venables and Ripley 2002) until a minimal adequate model was obtained. Contrasts between seed species and sowing rates from the field experiment were investigated using Tukey HSD post hoc tests implemented in the multcomp package (Hothorn et al. 2008). For the laboratory experiment, estimates with standard errors, *t*‐, and *P*‐values, as well as contrasts between factor levels were assessed from the summary table by reordering factor levels.

## Results

### Field experiment

**Figure 1:**
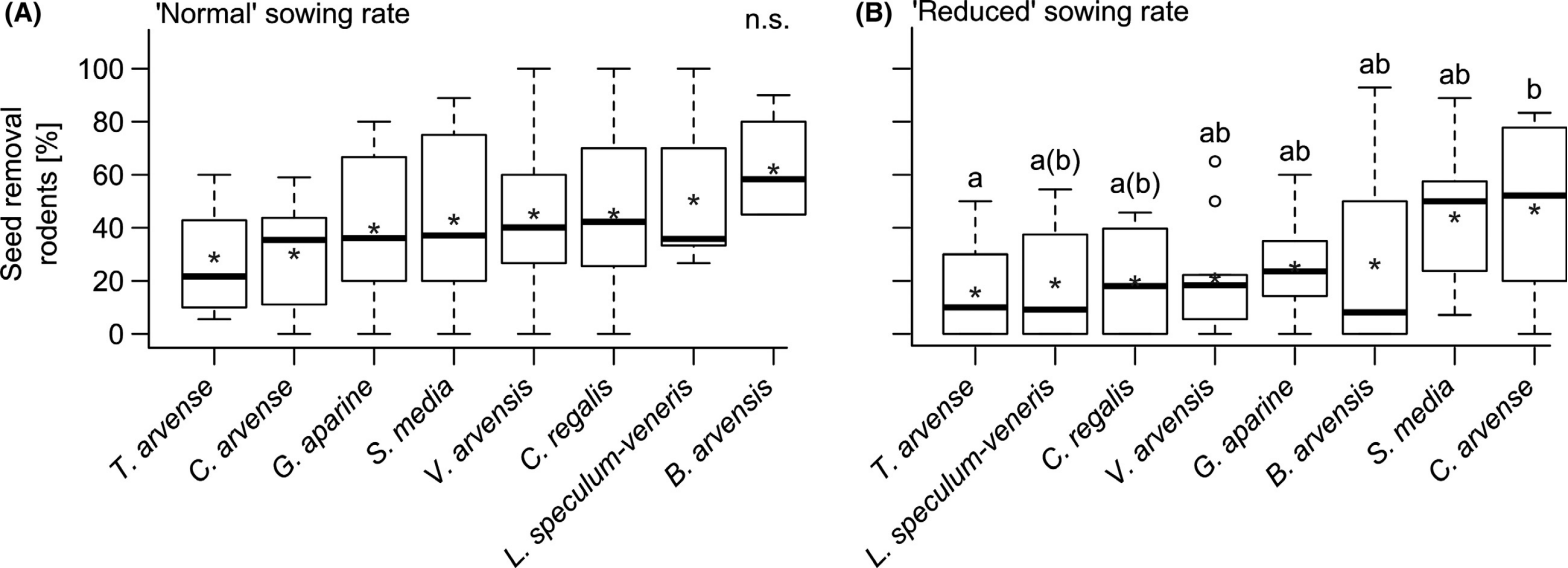
Boxplots of seed removal rates after 24 h in the field in plots with (A) “normal” sowing rates and (B) “reduced” sowing rates, tested for the different seed species. Seed species were sorted ascending by removal rates. Mean seed removal is indicated by “*”. Whiskers extend to the most extreme data point which is no more than 1.5 times the interquartile range from the box. Significant differences between seed species were derived from Tukey HSD post hoc tests implemented in the multcomp package. Plant species sharing the same letter are not significantly different from one another. Nonsignificant difference in seed removal rates among all seed species pairs is indicted by “n.s.”

Seed removal rates ranged from 5.56 ± 2.58% (mean ± SE) for *G. aparine* in the “no rodent access” treatment under “reduced” sowing rates to 65.26 ± 4.30% for *B. arvensis* in the “all access” treatment under “normal” sowing rates (Table S2). Overall SR_R_ in the field was 35.1 ± 2.2% after 24 h of exposition. SR_R_ was higher in “normal” sowing rates with 43.4 ± 3.0% compared to “reduced” sowing rates with 26.4 ± 2.9% (estimate ± SE: −17.5 ± 6.4, *t*_15_ = −2.7, *P* = 0.02). For *B. arvensis,* SR_R_ tended to be higher in plots with “normal” compared to “reduced” sowing rates (estimate ± SE: 35.8 ± 10.7, *z*_121_ = 3.4, *P* = 0.06; Table 1), while there was no difference in SR_R_ for the other seed species. In plots with “normal” sowing rates, there was no difference among the different seed species (Fig. 1A), while in plots with “reduced” sowing rates SR_R_ was lower for *T. arvense* compared to *C. arvense* (interaction: seed species × sowing rate; Fig. 1B). There was no difference in SR_R_ among seeds with different weight classes, nutrient content, or occurrence probability (Table 2).

**Table 2:** Results of linear mixed effects models showing effects on seed removal rates (SR_R_) in the field; species–specific seed predation rates (SP_R_) and Rodgers’ preference indices (*R*_i_) in the laboratory in relation to seed trait and plants’ occurrence probability. Mean values and parameter estimates with standard error (SE), degrees of freedom (df), *t*– and *P*–values are given. Bold values indicate significant differences in SR_R_/SP_R_ and *R*_i_ among factor levels of seed traits. Variables indicated by “–” were removed from the minimal adequate model

Experiment	Response variable	Species	Trait	Levels	SR_R_/SP_R_ (%)/*R*_i_	Model results			
						Estimate ± SE	df	*t*–Value	*P*–Value
Field	SR_R_		Weight	Light	33.9 ± 2.5	–	–	–	–
				Heavy	38.5 ± 4.4				
			Nutrients	1	33.3 ± 6.3	–	–	–	–
				0	35.4 ± 2.3				
			Red list	Endangered	37.3 ± 3.8	–	–	–	–
				Common	33.7 ± 2.7				
Laboratory	SP_R_	*M. arvalis*	Weight	Light	30.8 ± 3.6	–	–	–	–
				Heavy	33.9 ± 6.7				
			Nutrients	1	40.0 ± 6.1	12.8 ± 6.2^a^	83	2.1	**0.04**
				0	27.2 ± 3.6				
			Red list	Endangered	32.8 ± 7.5	–	–	–	–
				Common	31.1 ± 3.5				
		*M. glareolus*	Weight	Light	63.8 ± 2.6	40.0 ± 4.2^b^	279	9.6	**<0.001**
				Heavy	23.8 ± 3.6				
			Nutrients	1	59.9 ± 4.0	6.2 ± 2.9^a^	279	2.1	**0.04**
				0	53.8 ± 2.9				
			Red list	Endangered	45.0 ± 5.3	−13.5 ± 4.5^c^	279	−3.0	**0.003**
				Common	58.5 ± 2.6				
	*R*_i_	*M. arvalis*	Weight	Light	0.5 ± 0.0	–	–	–	**–**
				Heavy	0.5 ± 0.0				
			Nutrients	1	0.6 ± 0.0	0.1 ± 0.0^a^	83	2.2	**0.01**
				0	0.5 ± 0.0				
			Red list	Endangered	0.5 ± 0.1	–	–	–	–
				Common	0.5 ± 0.2				
		*M. glareolus*	Weight	Light	0.7 ± 0.0	0.3 ± 0.0^b^	279	10.7	**<0.001**
				Heavy	0.3 ± 0.0				
			Nutrients	1	0.6 ± 0.0	0.1 ± 0.0^a^	279	3.3	**0.001**
				0	0.6 ± 0.0				
			Red list	Endangered	0.5 ± 0.0	−0.1 ± 0.0^c^	279	−3.7	**<0.001**
				Common	0.6 ± 0.0				

a “0″ was the reference category.

b “heavy” was the reference category.

c “common” was the reference category.

Rodent abundances were very low with 10 individuals being captured on the experimental field (= 9.53 rodents·ha^−1^). The small rodent community consisted of *Apodemus flavicollis* (Melchior) (*n* = 4), *M. arvalis* (*n* = 3), and *Microtus agrestis* (L.), *M. glareolus*,* Apodemus sylvaticus* (L.) (*n* = 1, respectively).

In 720 video hours, rodents, but no other vertebrates such as birds, were observed to visit 40% of the seed depots (12 out of 30 observed depots) for 2275 sec., which equals 0.09% of the observation period. Thereby, rodents fed on seeds for 1341 sec. during 20 feeding events. Voles (*Microtus* spp. or *Myodes* sp.) spent with 390 sec. less time feeding on seeds and showed lower feeding diversity (*H* = 1.83) and evenness (*J* = 0.88) compared to mice (*Apodemus* spp.; 951 sec.; *H* = 2.05, *J* = 0.98). Voles preferred *B. arvensis* (30% feeding rate), while *L. speculum‐veneris* was never observed to be consumed. Mice preferred *B. arvensis* (16%) but also *G. aparine* (16%), while *S. media* (7%) were less often consumed (Table S3). In all cases where there was an interaction with the seeds, we observed rodents feeding on, but not removing seeds from seed depots (Fig. S2).

### Laboratory experiment

Overall SP_R_ in the laboratory was 50.2 ± 2.0% after 6 h and 93.2 ± 1.0% after 24 h of exposition. *Microtus arvalis* tended to consume less seeds after 6 h with 31.4 ± 3.2% compared to *M. glareolus* with 55.8 ± 2.4% (estimate ± SE: −24.4 ± 13.5, *t*_24_ = 1.80, *P* = 0.08).

**Figure 2:**
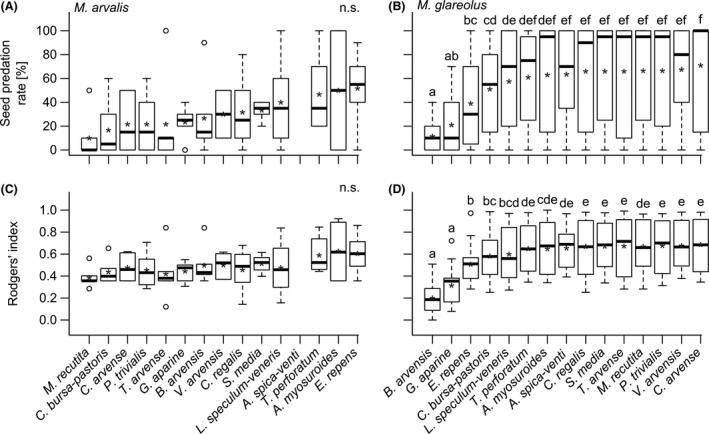
Boxplots of the seed predation rates in the laboratory after 6 h by (A) *M. arvalis* and (B) *M. glareolus,* and Rodgers’ preference index for (C) *M. arvalis* and (D) *M. glareolus* tested for the different seed species. Seed species were sorted ascending by seed predation rates per rodent species. Mean preference is indicated by “*”. Whiskers extend to the most extreme data point which is no more than 1.5 times the interquartile range from the box. Significance between seed species was assessed from the summary table of models by reordering factor levels. Plant species sharing the same letter are not significantly different from one another. Nonsignificant difference in preference among all seed species pairs is indicted by “n.s.”

For *M. arvalis,* SP_R_ as well as *R*_i_ of seeds containing nutrients were higher compared to nutrient‐poor seeds, while there was no difference among the different seed species (Table 1), weight classes, or occurrence probability (Fig. 2A,C, Table 2).

For *M. glareolus,* SP_R_ as well as *R*_i_ differed among the different seed species (Fig. 2B,D, Table 1). SP_R_ and *R*_i_ were higher for light compared to heavy seeds, for nutrient‐rich compared to nutrient‐poor seeds, and for common compared to endangered seed species (Table 2).

### Seed recovery after gut passage and germination

From the 150 seeds fed to each vole 0.2 ± 0.1 seeds·individual^−1^ were recovered in the feces samples. We found three seeds of *A. spica‐venti* and two *C. bursa‐pastoris* seeds (in feces of three voles). Just one of the *C. bursa‐pastoris* seeds germinated. Germination rates of undigested seeds differed among seed species (Table S4).

## Discussion

### Feeding preferences of rodents

In general, the selection and preference of food items depend on the size and physical and chemical properties of the food (Cousens et al. 2010; Maron et al. 2012). Results of the field experiment enabled us to draw conclusions about rodents’ seed feeding preferences and their biological control potential of weed seeds under natural conditions. Rodents’ feeding behavior in the field was seed species‐specific but not trait‐specific (weight, nutrient content). Under natural conditions, a variety of other seed predator guilds (mainly invertebrates) compete with rodents for seeds, depending on seed size (Fischer et al. 2011; but see Westerman et al. 2003). Therefore, it seems likely that rodents often feed on seeds which are remaining from other predators (e.g., the large‐seeded *B. arvensis,* for which seed predation by invertebrates may be constrained by their body mass; Honek et al. 2007); blurring their intrinsic feeding preferences, for example, for nutrient‐rich seeds. There was also no difference in seed removal rates depending on the occurrence probability of plants/seeds (endangered vs. common species). This can be explained by direct density‐dependent seed predation rates by rodents in agricultural fields (Baraibar et al. 2012; Schartel and Schauber 2016), because on our field site, the three endangered plant species abundantly occurred, besides the naturally occurring weeds, as they were intentionally planted for restoration approaches (Prestele et al. 2013). Furthermore, other factors such as a variety of different food items (other arable plant species and crops) within the field and in the surrounding landscape (Wilson et al. 1999), and the occurrence of other seed predators also influence rodents’ seed feeding preferences under natural conditions (Cousens et al. 2010).

The complementary laboratory experiment enabled us to examine intrinsic feeding preferences of rodents by offering seeds as the sole food source. Here, we found preferences for nutrient‐rich compared to nutrient‐poor seeds for *M. arvalis*, as well as for *M. glareolus* (c.f. Wang and Yang 2014). Thereby, seed predation rates by *M. arvalis* were marginally significantly lower compared to *M. glareolus,* and *M. arvalis* showed no preferences for seeds depending on seed species, weight, or occurrence probability*. Microtus arvalis* is an opportunistic feeder and has no preferences for grains with different protein and fiber content (Heroldová et al. 2008). In general, it feeds on low‐energy green plant material and rarely on seeds (3.9 ± 10% of its diet, *n* = 3; Butet and Delettre 2011), depending on the season (Hoogenboom et al. 1984). In contrast, *M. glareolus* feeds on a diversity of food sources, including different plant material, animal food, and seeds (25.9 ± 3.3%, *n* = 20; Butet and Delettre 2011). *Myodes glareolus* showed a preference for lightweight compared to heavy seeds. In accordance with the optimal foraging theory, feeding preferences are a trade‐off between energy intake and handling time (MacArthur and Pianka 1966). In our study, two of the three heavy seeds were nutrient‐poor, and feeding on larger seeds normally requires longer handling time (Wang and Yang 2014). Thus, a preference for lightweight seed species would reduce handling time and therefore may maximize the net energy intake, especially when searching time is minimized under controlled conditions in the laboratory. Further, *M. glareolus* preferred seeds of common over those of endangered plant species. This could be explained by a memory of food choice (Galef and Giraldeau 2001). *Myodes glareolus* has a better spatial learning ability compared to *M. arvalis* (Haupt et al. 2010) and may therefore prefer seeds they are familiar with, such as common plant species, which can occur in higher densities in agricultural fields and cause serious yield loss (Hofmeister and Garve 2006; Gehring and Thyssen 2011).

Using numerical seed traits, such as diaspore mass or actual protein and oil content, rather than grouping them into broad categories, may lead to better predictions of food selection by rodents (Cousens et al. 2010), but this approach was limited in our study for statistical reasons and due to limited trait data availability. Nevertheless, our results bring some advance in the understanding of rodent seed choice compared to other studies, which often do not include any seed traits or are not using a multiple‐species approach in seed predation studies (e.g., Meiss et al. 2010; Baraibar et al. 2012; Daedlow et al. 2014; but see Wang and Yang 2014). Future studies should manipulate, for instance, nutritional value of seeds, either using seed dummies or within species variability in nutrients of seeds, where nutritional value is the only variable trait. Further, field studies need to take density‐dependent food choice into account, to reliably predict the survival of rare plant species and the biological control of common plant species in relation to rodents’ seed predation rates.

### Endozoochorous seed dispersal and post‐dispersal seed predation

Our study shows that endozoochorous seed dispersal by voles can virtually be neglected. We further confirmed the role of small rodents as post‐dispersal seed predators rather than seed dispersers in agricultural fields by proximal camera observations (c.f. Vander Wall et al. 2005), where all observed voles and mice fed on seeds but did not transport seeds away from the depot (no indication for synzoochory). This results in seed predation rates of 35% in 24 h in the field, even if rodent abundance was very low, with 10 individuals·ha^−1^. If seeds are exposed over a longer period of time, and if higher rodent abundances prevail, predation rates can further increase in agricultural fields (Daedlow et al. 2014). Thereby, environmental factors, such as vegetation density, which led to higher seed removal rates in our field experiment on plots with “normal” sowing rates due to higher vegetation cover compared to plots with “reduced” sowing rates, may further reduce the seed input into the soil seedbank.

In terms of voles’ ecosystem functions in agricultural landscapes, results suggest that *M. glareolus* and to a lower extent *M. arvalis* can provide regulating ecosystem services as seed predators leading to biological weed control (c.f. Westerman et al. 2003; Daedlow et al. 2014). In particular in the case of often herbicide resistant grass species, such as *A. myosuroides* (Gehring and Thyssen 2011), seed predation rates of up to 50% in 6 h (shown in the laboratory experiment) and intrinsic feeding preferences of voles may reduce dispersal and proliferation of these noxious weeds in different kind of crops. Seed predation by the community of seed predators, including voles, but also *Apodemus* spp., which fed on the seeds in our field experiment nonselectively, as well as larger insects (Menalled et al. 2007; Fischer et al. 2011; but see Westerman et al. 2003) can affect the demography of plants and limit their recruitment in agricultural landscapes. As *M. glareolus* mainly occurs in habitat edges, but not in crop fields (Heroldová et al. 2007; Fischer and Schröder 2014), it may restrict the spillover of weed seeds from edges into fields. Seed predation by the open‐land species *M. arvalis* (Heroldová et al. 2007), in contrast, may reduce within field weed densities. However, as voles can reach high population densities due to distinct population cycles (Cornulier et al. 2013), not only may biological control of weeds increase, but also crop yield may be negatively affected to a higher degree. Therefore, further studies on seed predation rates and the biological control potential, which can be highly variable in space and time according to fluctuating vole densities, need to take these spatial and temporal variations into account.

## Conclusion

Seeds are valuable food items for small rodents in agricultural landscapes due to their high nutrient content (Butet and Delettre 2011). At a time of high input farming and increasing herbicide resistance of weeds, post‐dispersal seed predation on common weeds can provide regulating ecosystem services in terms of biological weed control (Westerman et al. 2003; Daedlow et al. 2014). On the other hand, seed dispersal can lead to farmland restoration by spreading endangered arable plants (Benayas and Bullock 2012). Our study shows that voles are rather seed predators than seed dispersers and may function as biological weed control agents of common weeds. The risk of affecting endangered arable plants by further depleting the seedbank seems to be low, as voles showed an intrinsic feeding preference for common compared to endangered seeds species, which might be related to local adaption to food availability and spatial learning ability (Galef and Giraldeau 2001; Haupt et al. 2010), and therefore, seed predation by rodents may act as a biotic filter for noxious weeds (Myers and Harms 2009). Under natural conditions in agricultural fields, however, predation rates and feeding preferences of other seed predators, such as *Apodemus* spp., as well as factors, such as vole population densities (Cornulier et al. 2013), animal movement (Cousens et al. 2010), field management, and landscape scale effects (Fischer et al. 2011), need to be taken into account to reliably predict the impact of rodents on the seed fate of arable plants.

## Conflict of Interest

None declared.

## Supporting information


**Table S1.** Species information about arable plant species.
***Table S2.** Seed removal rates in the field for the different plant species, sowing rates and predator guilds.*
***Table S3.** Feeding rate by mice and voles from video observations in the field.*
***Table S4.** Germination rates of arable plant species.*
***Figure S1.** Arrangement of the seed removal experiment and the rodent traps in the experimental field.*
**Figure S2.** *Myodes glareolus* and *Apodemus* spp. feeding on seeds.
