# Supplementary material for: Seed preferences by rodents in the agri‐environment and implications for biological weed control
Source: Ecol Evol. 2016 Jul 22;6(16):5796–807. doi: 10.1002/ece3.2329 (PMC4983592; doi:10.1002/ece3.2329)
Supplement: Supplementary file 1 — Table S1. Species information about arable plant species. Table S2. Seed removal rates in the field for the different plant species, sowing rates and predator guilds. Table S3. Feeding rate by mice and voles from video observations in the field. Table S4. Germination rates of arable plant species. Figure S1. Arrangement of the seed removal experiment and the rodent traps in the experimental field. Figure S2. Myodes glareolus and Apodemus spp. feeding on seeds. [file ECE3-6-5796-s001.docx]

**Supporting Information**

**Table S1.** Species information about arable plant species (nomenclature according to Wisskirchen & Haeupler 1998) which were used for the seed removal/ predation experiment in the field and in the laboratory, with common names, plants’ reproduction type and seed weight.

| **Plant species** | **Common name** | **Type of reproduction*** | **Weight (mg)** |
| --- | --- | --- | --- |
| *Alopecurus myosuroides* Huds. | Slender meadow foxtail | by seed | 2.10† |
| *Apera spica-venti* (L.) P. Beauv | Silky bent grass | by seed | 0.02‡ |
| *Buglossoides arvensis* (L.) I. M. Johnst. | Field gromwell | by seed | 5.74‡ |
| *Capsella bursa-pastoris* (L.) Med. | Shepherd’s purse | by seed | 0.13† |
| *Cirsium arvense* (L.) Scop. | Creeping thistle | by seed and vegetatively | 0.84‡ |
| *Consolida regalis* Gray | Gray Forking larkspur | by seed | 1.26‡ |
| *Elymus repens* (L.) Gould s. str | Cough grass | mostly vegetatively, rarely by seed | 7.23† |
| *Galium aparine* L. | Cleavers | by seed | 6.00‡ |
| *Legousia speculum-veneris* (L.) Chaix | Venus’ looking glass | by seed | 0.21‡ |
| *Matricaria recutita* L. | German chamomile | by seed | 0.14¶ |
| *Poa trivialis* L. s.l. | Rough bluegrass | by seed and vegetatively | 0.02‡ |
| *Stellaria media* (L.) Vill. S.str. | Common chickweed | by seed | 0.39‡ |
| *Thlaspi arvense* L. | Field penny-cress | by seed | 0.97‡ |
| *Tripleurospermum perforatum* (Mérat) Lainz | Scentless chamomile | by seed | 0.39† |
| *Viola arvensis* Murray | Field pansy | by seed | 0.61‡ |

* Plant characteristics extracted from the BiolFlor database (Klotz, Kühn & Durka 2002)

† Traits extracted from the D^3^ database (Hintze *et al.* 2013)

‡ Own measurements

¶ Traits extracted from the SID database (Royal Botanic Gardens Kew 2015)

**Table S2.** Mean seed removal rates with SE for the different seed species, sowing rates of rye and predator exclusion treatments in the field experiment.

| **Plant species** | **‘normal’ sowing rate** | | **‘reduced’ sowing rate** | |
| --- | --- | --- | --- | --- |
|  | **all access** | **no rodent access** | **all access** | **no rodent access** |
| *Buglossoides arvensis* (L.) I. M. Johnst. | 65.26 ± 7.81 | 12.11 ± 4.30 | 33.33 ± 9.29 | 10.56 ± 4.08 |
| *Cirsium arvense* (L.) Scop. | 49.47 ± 6.47 | 27.89 ± 5.22 | 58.00 ± 9.17 | 18.00 ± 3.55 |
| *Consolida regalis* Gray | 48.13 ± 8.62 | 15.00 ± 5.92 | 26.11 ± 7.05 | 8.89 ± 2.27 |
| *Galium aparine* L. | 42.94 ± 8.44 | 16.47 ± 5.62 | 26.11 ± 5.55 | 5.56 ± 2.58 |
| *Legousia speculum-veneris* (L.) Chaix | 62.94 ± 5.34 | 29.41 ± 5.18 | 40.63 ± 5.95 | 23.75 ± 3.97 |
| *Stellaria media* (L.) Vill. S.str. | 63.33 ± 6.37 | 35.33 ± 5.15 | 53.33 ± 11.30 | 20.00 ± 5.77 |
| *Thlaspi arvense* L. | 33.33 ± 7.47 | 12.67 ± 3.58 | 22.50 ± 6.55 | 8.75 ± 2.39 |
| *Viola arvensis* Murray | 54.67 ± 6.75 | 17.33 ± 4.92 | 36.67 ± 6.86 | 25.56 ± 7.20 |
| **Mean** | **52.51** ± **7.16** | **20.78** ± **4.99** | **37.09** ± **7.72** | **15.13** ± **3.98** |

**Table S3.** Feeding rate, calculated from the total number of seed feeding events, of voles (including *Microtus* spp. and *Myodes* sp.) and mice (*Apodemus* spp.) observed by video recording in the field.

| **Seed species** | **Feeding rate (%)** | |
| --- | --- | --- |
|  | **Voles** | **Mice** |
| *B. arvensis* | 30 | 16 |
| *C. arvense* | 10 | 14 |
| *C. regalis* | 10 | 12 |
| *G. aparine* | 10 | 16 |
| *L. speculum-veneris* | 0 | 12 |
| *S. media* | 10 | 7 |
| *T. arvense* | 10 | 9 |
| *V. arvensis* | 20 | 14 |

**Table S4.** Germination rates of arable plant species with standard errors (SE) after 6 weeks. Germination rates differed among seed species (F_14_ = 98.80, p< 0.001; permutational ANOVA implemented in the R package RVAideMemoire (Hervé 2015).

| **Seed species** | **Germination rate ± SE (%)** |
| --- | --- |
| *A. myosuroides* | 87.5 ± 3 |
| *A. spica-venti* | 2.5 ± 3 |
| *B. arvensis* | 85.0 ± 3 |
| *C. bursa-pastoris* | 6.3 ± 1 |
| *C. arvense* | 3.8 ± 1 |
| *C. regalis* | 17.5 ± 3 |
| *E. repens* | 61.3 ± 9 |
| *G. aparine* | 56.3 ± 1 |
| *L. speculum-veneris* | 65.0 ± 3 |
| *M. recutita* | 68.8 ± 1 |
| *P. trivialis* | 41.3 ± 4 |
| *S. media* | 65.0 ± 5 |
| *T. arvense* | 6.3 ± 1 |
| *T. perforatum* | 95.0 ± 3 |
| *V. arvensis* | 36.3 ± 1 |

**Fig. S1.** Arrangement of the seed removal experiment with the two different predator exclusion treatments and rodent traps within the experimental crop field, consisting of five replications with 16 plots each. Per replication, two plots were randomly selected for the seed removal experiment planted with winter rye with normal sowing rate (350 seed/m²) and two plots with winter rye with reduced sowing rate (88 seed/m²).

**
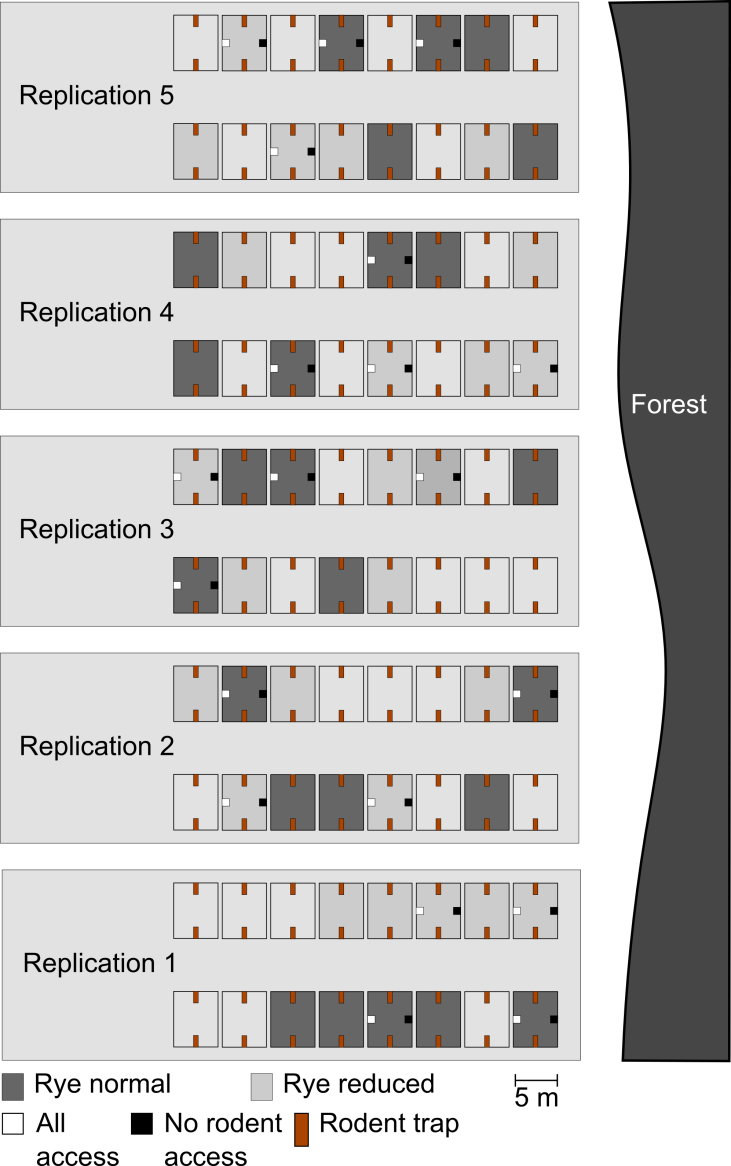
**

**Fig. S2.** Results of proximal camera observations from the field experiment, showing *Myodes* *glareolus* (a) and *Apodemus* spp. (b) feeding on seeds from our seed depot.

| (a) | 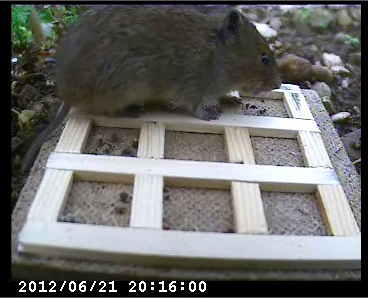 |
| --- | --- |
| (b) | 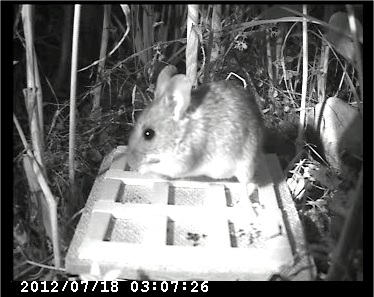 |

Hervé, M. (2015) RVAideMemoire: Diverse Basic Statistical and Graphical Functions. R package version 0.9-45-2. http://CRAN.R-project.org/package=RVAideMemoire

Hintze, C., Heydel, F., Hoppe, C., Cunze, S., Konig, A. & Tackenberg, O. (2013) D^3^: The Dispersal and diaspore database -Baseline data and statistics on seed dispersal. Perspect. Plant Ecol. Evol. Syst. 15:180–192.

Klotz, S., Kühn, I. & Durka, W. (2002) *BIOLFLOR - Eine Datenbank zu biologisch-ökologischen Merkmalen der Gefäßpflanzen in Deutschland*. Bundesamt für Naturschutz, Bonn.

Royal Botanic Gardens Kew (2015) Seed Information Database (SID). Version 7.1.

Wisskirchen, R. & Haeupler, H. (1998) *Standardliste der Farn- und Blütenpflanzen Deutschlands*. Ulmer, Stuttgart
